# Conservation of Cdc14 phosphatase specificity in plant fungal pathogens: implications for antifungal development

**DOI:** 10.1038/s41598-020-68921-3

**Published:** 2020-07-21

**Authors:** Andrew G. DeMarco, Kedric L. Milholland, Amanda L. Pendleton, John J. Whitney, Peipei Zhu, Daniel T. Wesenberg, Monessha Nambiar, Antonella Pepe, Stefan Paula, Jean Chmielewski, Jennifer H. Wisecaver, W. Andy Tao, Mark C. Hall

**Affiliations:** 1 Department of Biochemistry, Purdue University, West Lafayette, IN 47907 USA; 2 Department of Chemistry, Purdue University, West Lafayette, IN 47907 USA; 3 Center for Cancer Research, Purdue University, West Lafayette, IN 47907 USA; 4 Center for Plant Biology, Purdue University, West Lafayette, IN 47907 USA; 5 Present Address: Institute of Chemical Biology and Drug Discovery, Stony Brook University, Stony Brook, NY 11794-3400 USA; 6 Present Address: Department of Chemistry, California State University, 6000 J Street, Sacramento, CA 95819 USA

**Keywords:** Analytical biochemistry, Mass spectrometry, Phylogenetics, Enzymes, Fungi, Pathogens

## Abstract

Cdc14 protein phosphatases play an important role in plant infection by several fungal pathogens. This and other properties of Cdc14 enzymes make them an intriguing target for development of new antifungal crop treatments. Active site architecture and substrate specificity of Cdc14 from the model fungus *Saccharomyces cerevisiae* (ScCdc14) are well-defined and unique among characterized phosphatases. Cdc14 appears absent from some model plants. However, the extent of conservation of Cdc14 sequence, structure, and specificity in fungal plant pathogens is unknown. We addressed this by performing a comprehensive phylogenetic analysis of the Cdc14 family and comparing the conservation of active site structure and specificity among a sampling of plant pathogen Cdc14 homologs. We show that Cdc14 was lost in the common ancestor of angiosperm plants but is ubiquitous in ascomycete and basidiomycete fungi. The unique substrate specificity of ScCdc14 was invariant in homologs from eight diverse species of dikarya, suggesting it is conserved across the lineage. A synthetic substrate mimetic inhibited diverse fungal Cdc14 homologs with similar low µM *K*_*i*_ values, but had little effect on related phosphatases. Our results justify future exploration of Cdc14 as a broad spectrum antifungal target for plant protection.

## Introduction

Plant pathogens pose a constant threat to agricultural productivity and global food security, with fungi and the fungal-like oomycetes being the most dangerous culprits^1–4^. Despite the development of chemical pesticides and disease-resistant cultivars to curb crop infections over the past century, damage from fungal and other pathogens persists at nearly comparable levels^3^. Estimates suggest more than 10% of the world agricultural harvest may be lost annually to fungal infections alone, equating to hundreds of billions of dollars and enough food to feed an estimated 600 million people^2–5^. Post-harvest losses from fungal-induced spoilage and toxin accumulation further exacerbate the problem, especially in developing countries^6^. A major challenge to effectively suppressing fungal crop diseases is the ability of fungi to rapidly develop resistance to pesticides and acquire mutations that counteract plant defenses in disease-resistant lines^2,3,7,8^. Consequently, the continual battle against fungal pathogens requires a constant stream of new management strategies, including both the generation of new infection resistance mechanisms in crops along with identification of novel pesticide compounds and targets^1^.

The Cdc14 phosphatases, known best for roles in counteracting cyclin-dependent kinase activity during mitosis in model fungi like *Saccharomyces cerevisiae* and *Schizosaccharomyces pombe*^9,10^ may be an attractive novel target for development of broad-acting antifungal agents. Deletion of the *CDC14* gene in several plant pathogen species severely impairs virulence, demonstrating that Cdc14 function is important for host infection^11–13^. *Fusarium graminearum* lacking *CDC14* exhibited defective conidia and ascospore formation and was unable to infect and colonize wheat heads, despite only a modest reduction in vegetative growth^11^. *Magnaporthe oryzae* lacking *CDC14* showed similar phenotypes characterized by severely reduced conidiation, defective appressoria formation, and ineffective leaf penetration and infection^12^. Deletion of *CDC14* in *Aspergillus flavus* greatly reduced conidiation and pathogenicity in a seed infection assay but had minimal impact on vegetative growth rate^13^. A common cellular phenotype associated with *CDC14* deletion in these studies was defective cytokinesis/septation and coordination with nuclear division. A similar phenotype coupled with defective conidiation and reduced virulence was observed upon *CDC14* deletion in the entomopathogenic fungus *Beauveria bassiana*^14^, and *CDC14* deletion in the opportunistic human pathogen *Candida albicans* resulted in cytokinesis defects and reduced hyphal growth required for infection^15^. Even in the oomycete *Phytopthora infestans,* Cdc14 is required for generation of asexual infectious spores^16^. Thus, in fungi and oomycetes, Cdc14 seems to promote host infection and, by extension, inhibition of Cdc14 could help prevent infections. Mechanistic details of how Cdc14 contributes to infection, including the identification of relevant substrates, are still lacking.

Several other features of Cdc14 make it an attractive antifungal target, in principle. First, *CDC14* may be absent in most land plant genomes based on similarity searching of a handful of model plant genome sequences^17,18^. Second, deletion of CDC14 genes in several model animal systems had little to no impact on cell division and development^19–24^. In general, Cdc14 functions are thought to be poorly conserved between animals and fungi^25^, despite the apparently high conservation of Cdc14 structure between these lineages^26,27^. Thus, treatments targeting Cdc14 might be predicted to have little adverse effect on plants or on animals consuming treated plant products.

Third, the structure and specificity of the Cdc14 active site may be conducive to development of highly selective inhibitors. The Cdc14 family is structurally and mechanistically related to the dual specificity phosphatase (DSP) subfamily of protein tyrosine phosphatases (PTPs), characterized by the invariant HCX_5_R active site motif with catalytic cysteine^26,28–31^. However, biochemical characterizations revealed that *S. cerevisiae* Cdc14 (ScCdc14) evolved to act very specifically on phosphoserine substrates of proline-directed kinases (pSer-Pro), most notably cyclin-dependent kinases^32–34^, a property that appears conserved in human Cdc14A and Cdc14B^32^. ScCdc14 further requires a basic amino acid, preferably Lys, at the + 3 position relative to pSer for efficient catalysis both in vitro and in vivo^33,34^. Optimal substrates have additional basic amino acids around the + 3 position^33^. *F. graminearum* Cdc14 exhibits similar substrate preference^11^, but specificity has not been characterized in other plant pathogen Cdc14 homologs.

The structural basis for recognition of the core pSer-Pro-x-Lys substrate motif by the ScCdc14 active site region is understood^27,33^ and will be useful in the optimization of inhibitor structures. The strict substrate specificity of the Cdc14 catalytic core contrasts with that of most Ser/Thr phosphatases, including the ubiquitous phosphoprotein phosphatase family members PP1 and PP2A, which consist of relatively un-specific catalytic subunits associated with substrate-recruiting accessory factors^35^. Specific inhibitor development has been challenging for many Ser/Thr phosphatases^36,37^.

For Cdc14 to be an effective and broad-acting antifungal target, it should be ubiquitous in plant fungal pathogen species, and its structure and enzymatic specificity should be highly conserved, thus providing a common, well-defined target site for inhibitor binding. Here, we globally assessed the phylogenetic distribution of Cdc14 in eukaryotes and the conservation of Cdc14 structure and substrate specificity in diverse fungal plant pathogens. The results provide support for this enzyme family being pursued as a novel antifungal target.

## Results

### *CDC14* is broadly conserved in plant fungal pathogens but absent from angiosperms

**Figure 1:**
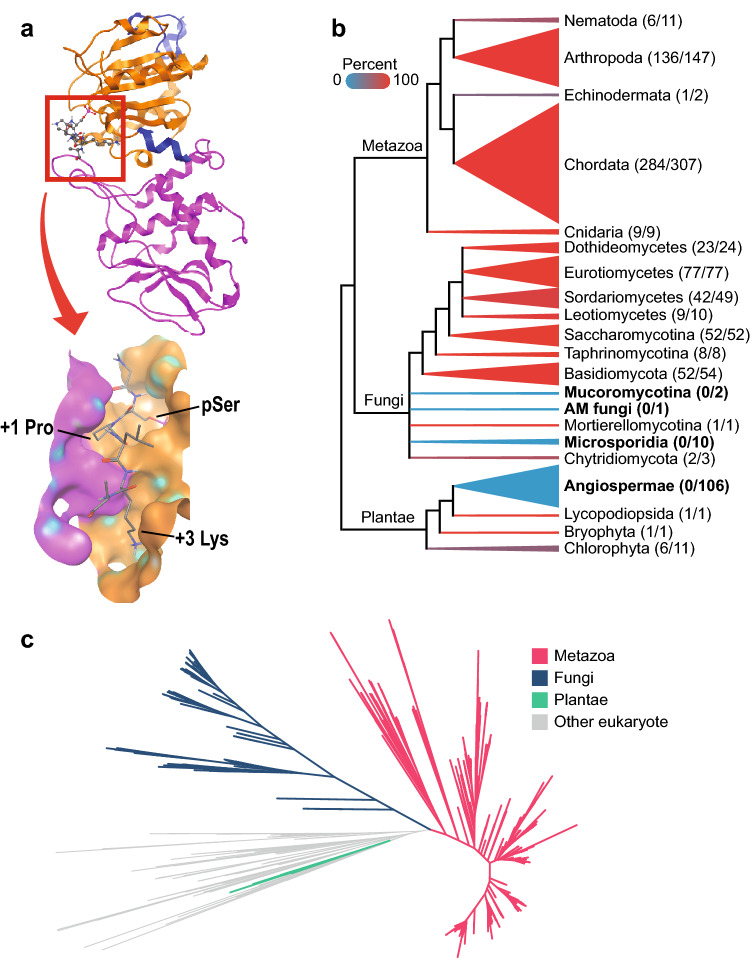
Phylogenetic analysis of Cdc14. (**a**) Top, ribbon structure of the ScCdc14 catalytic domain (PDB 5XW5) with bound peptide substrate in ball and stick representation (red box). The N-terminal DSPn domain is colored magenta and the C-terminal DSPc domain containing the catalytic motif orange. The boxed region is enlarged in surface representation below showing the substrate binding site with key substrate recognition features labeled. (**b**) Taxonomic distribution of CDC14 gene family across Metazoa, Fungi, and Plantae. The height of the triangles indicates the relative representation of each lineage in the database, and the color of the triangles indicates the percentage of each lineage found to contain at least one copy of CDC14. The numbers to the right of each lineage name are the number of species containing one or more CDC14 gene/species in the database. The lineages in which no CDC14 copies were identified are bolded. (**c**) Unrooted maximum likelihood phylogenetic tree of Cdc14 genes.

In previous studies, *CDC14* homologs were found in green algae, bryophytes, and lycophytes, but not in the model angiosperms *A. thaliana, O. sativa,* and *P. trichocarpa*^17,18^. While *CDC14* has been studied in model fungal species and a handful of fungal pathogens, the phylogenetic distribution of *CDC14* in the fungal kingdom has not been systematically characterized with the abundant genome sequence data currently available. We used HMMER to identify homologs of ScCdc14 in the NCBI RefSeq database of protein sequences from nearly 27,000 taxa (Supplementary Table S1 online) and evaluate its conservation across plant, fungal, and other eukaryotic lineages. A unique structural feature of Cdc14 enzymes is the presence of two dual specificity phosphatase domains. The N-terminal domain is non-catalytic but contributes critically important residues to the active site, which sits at an interface between the two conserved domains^26,27^ (Fig. 1a). This atypical arrangement likely accounts for the unique specificity of Cdc14 enzymes and we therefore used it as the primary criterion for distinguishing true Cdc14 homologs from other related DSPs and PTPs. We further required that reciprocal BLAST searches with candidate homolog sequences against *S. cerevisiae* and *H. sapiens* return Cdc14 as the best hits. A compiled list of species containing Cdc14 homologs is presented in Supplementary Table S2 online. A full list of the identified Cdc14 homolog sequences and BLAST confirmation results is presented in Supplementary Table S3 online.

Cdc14 homologs were abundant in metazoan and fungal lineages but, consistent with the previous reports, were not observed in any angiosperm plant species. To rigorously assess the presence and absence of Cdc14 genes in different eukaryotic lineages we limited the RefSeq database to fungal, animal, and green plant (i.e. Plantae or Viridiplantae) species with a minimum of 1,800 protein sequences. This filtered database contained 106 angiosperms, yet no significant protein matches to Cdc14 were found (Fig. 1b and Supplementary Fig. S1, File S1, and Table S4 online). Among land plants in the filtered database, a single homolog of Cdc14 was found in the bryophyte *Physcomitrella patens* and the lycophyte *Selaginella moellendorffi*, also consistent with the prior reports. Of the 11 green algal (i.e. Chlorophyta) species in the filtered database, 6 contained one homolog of Cdc14 each. Collectively, this analysis provides strong confirmation that Cdc14 genes were lost in a common ancestor of angiosperms. Within the fungal kingdom, Cdc14 homologs were nearly ubiquitous in Basidiomycota and Ascomycota, which contain the majority of plant fungal pathogens. Interestingly, a Cdc14 homolog was not observed in *Rhizophagus irregularis*, a glomeromycete that forms beneficial nutrient-harvesting arbuscular mycorrhizas in plant root systems. Further BLAST searches against additional public glomeromycete genomes failed to retrieve any Cdc14 homologs, suggesting that Cdc14 is absent from this fungal branch. In general, fungal Cdc14 homologs were sparse outside the Dikarya.

A maximum likelihood phylogeny of the Cdc14 gene family shows fungal Cdc14 homologs grouping sister to Cdc14 homologs from metazoans as expected based on the eukaryotic tree of life (Fig. 1c). The few green algal and plant Cdc14 homologs grouped with sequences from other eukaryotes, but with weak branch support (Supplementary Fig. S1 and File S1 online). Although many metazoans contain multiple Cdc14 genes, the majority of fungal species contain only a single Cdc14 gene. A few eukaryotes appear to have expanded this gene family, exemplified by the Cryptophyte *Guillardia theta*, which contains 16 database entries homologous to Cdc14 (Supplementary Table S2 online).

### The Cdc14 active site is invariant across fungal pathogens

**Figure 2:**
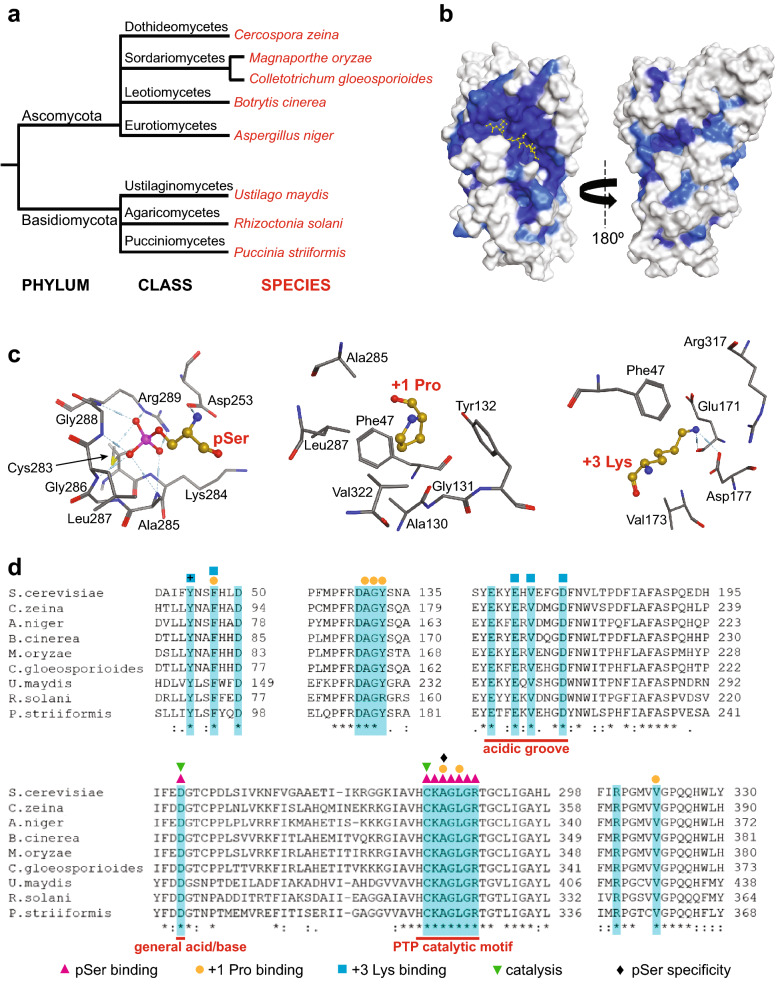
The Cdc14 active site is highly conserved in plant pathogenic fungi. (**a**) Fungal lineages of the eight plant pathogen species chosen for study. (**b**) Structural model of ScCdc14 (PDB 5XW5) colored to map sequence conservation among the eight plant pathogen homologs from (**a**). Dark blue indicates identity, and light blue similarity, in all species. The yellow ball and stick phosphopeptide substrate molecule marks the active site and substrate binding groove location. (**c**) ScCdc14 amino acids within 4.5 Å of the substrate pSer, + 1 Pro, and + 3 Lys in structure 5XW5. Dashed lines between atoms indicate hydrogen bonds. (**d**) Clustal Omega alignment of the plant pathogen Cdc14 homologs from (**a**) with ScCdc14. Highlighted residues include those shown in (**c**), and others previously implicated in substrate recognition and specificity^26,31–33^. Colored symbols above highlighted residues indicate substrate feature(s) that each is known or predicted (+) to interact with.

Human Cdc14B and ScCdc14 share very similar active sites^26,27^ and at least some substrate specificity features^32^. This suggests that Cdc14 structures and substrate preferences may be broadly conserved across eukaryotes. To explore this further, we selected eight Cdc14 homologs from plant pathogens representing diverse groups within the phyla Ascomycota and Basidiomycota (Fig. 2a), aligned their primary sequences with ScCdc14 (Supplementary Fig. S2 online), and then mapped the extent of sequence conservation onto ScCdc14 structure 5XW5^27^, which contains a bound phosphopeptide from the ScCdc14 substrate Swi6 (Fig. 2b). The active site region containing the bound phosphopeptide substrate is nearly invariant in the aligned sequences, whereas sparse conservation is observed across the rest of the structure. Of the four substrate recognition determinants defined biochemically with ScCdc14^32,33^ and described in the introduction, the structural basis for the first three are readily observed in the 5XW5 ScCdc14-substrate structure. Figure 2c highlights the amino acids within the active site and substrate binding groove that are in proximity (< 4.5 Å) to these critical substrate features. We next specifically considered the conservation of these and other amino acid residues known or predicted to contribute to substrate binding (Fig. 2d). The multisequence alignment reveals that every amino acid known or predicted to contribute to substrate binding and catalysis is invariant across these diverse fungal species, with one exception. *R. solani* Cdc14 (RsCdc14) contains an arginine in the position equivalent to Tyr132 of ScCdc14 and all the other Cdc14 homologs. This Tyr residue appears to form a “cap” over the apolar cavity that accommodates the + 1 Pro sidechain (Fig. 2c). The striking conservation of the substrate binding regions of fungal Cdc14 enzymes suggests that substrate specificity may also be highly conserved.

### The activity and substrate specificity of Cdc14 homologs in plant pathogenic fungi is highly conserved

All of the fungal Cdc14 homologs aligned in Fig. 2d were recombinantly expressed in *E. coli* with N-terminal 6×-histidine tags and purified by nickel affinity chromatography (Supplementary Fig. S3 online). We compared the steady-state kinetic parameters *k*_cat_ and *K*_M_ of each fungal pathogen enzyme with ScCdc14 using the small molecule phospho compounds *para*-nitrophenyl phosphate (pNPP) and 6,8-difluoro-4-methylumbelliferyl phosphate (DiFMUP), and a phosphopeptide sequence derived from the known ScCdc14 substrate site, Yen1 serine 583^33^. The kinetic parameters for ScCdc14 were in reasonably good agreement with previously reported values^31^ considering reaction condition differences. Although there was modest variation among the homologs, the kinetic parameters for the different substrates followed strikingly similar trends (Table 1). As previously shown for ScCdc14^31^, DiFMUP was a much better substrate than pNPP for all homologs, with *k*_*cat*_*/K*_*M*_ consistently 3–4 orders of magnitude higher. This difference derived primarily from much lower *K*_*M*_ values for DiFMUP. Despite the wide variations in *K*_*M*_ between the three substrates, the *k*_*cat*_ values were similar, consistent with the idea that hydrolysis of the phosphoenzyme intermediate is the rate-limiting catalytic step for all homologs^30^. These experiments demonstrate that Cdc14 homologs from diverse fungal species possess similar, though not identical, catalytic properties, as would be expected from the strict evolutionary conservation of their active sites.

**Table 1:** Steady-state kinetic parameters for fungal pathogen Cdc14 homologs.

Enzyme^a^	pNPP			DiFMUP			Phosphopeptide pS^b^		
	*k*_cat_ (s^−1^)	*K*_M_ (mM)	*k*_cat_/*K*_M_ (M^−1^ s^−1^)	*k*_cat_ (s^−1^)	*K*_M_ (µM)	*k*_cat_/*K*_M_ (M^−1^ s^−1^)	*k*_cat_ (s^−1^)	*K*_M_ (µM)	*k*_cat_/*K*_M_ (M^−1^ s^−1^)
ScCdc14	1.12 ± 0.08	23 ± 1	50 ± 4	3 ± 0.4	11 ± 3	253,000 ± 34,000	1.4 ± 0.1	173 ± 21	7,800 ± 300
AnCdc14	0.73 ± 0.03	35 ± 2	21 ± 1	3 ± 0.2	36 ± 4	83,000 ± 9,400	1.0 ± 0.1	194 ± 44	5,100 ± 800
BcCdc14	0.380 ± 0.003	16 ± 2	24 ± 2	1 ± 0.2	34 ± 7	43,100 ± 11,000	1.0 ± 0.1	297 ± 38	3,400 ± 300
CgCdc14	0.6 ± 0.1	30 ± 4	20 ± 2	7 ± 0.9	78 ± 12	86,000 ± 6,100	2.2 ± 0.1	351 ± 21	6,200 ± 200
CzCdc14	2.8 ± 0.1	25 ± 2	115 ± 6	31 ± 2	66 ± 11	477,000 ± 10,600	2.1 ± 0.1	62 ± 14	34,000 ± 8,000
MoCdc14	1.02 ± 0.03	25 ± 1	40 ± 2	3 ± 0.5	44 ± 11	68,000 ± 6,100	0.4 ± 0.02	63 ± 19	6,200 ± 1,700
PsCdc14	0.12 ± 0.02	29 ± 3	12 ± 1	0.21 ± 0.01	4.9 ± 0.9	45,500 ± 6,200	ND^**c**^	ND	ND
RsCdc14	0.61 ± 0.04	18 ± 2	23 ± 2	1 ± 0.1	19 ± 7	35,000 ± 8,500	0.52 ± 0.07	178 ± 52	3,000 ± 600

All kinetic parameters were determined by fitting the Michaelis–Menten equation to velocity vs. substrate concentration plots in GraphPad Prism. Each value represents the mean of 3 independent experiments ± standard deviations.

UmCdc14 (*Ustilago maydis)* was not subjected to steady-state kinetic analysis because its truncation removed a conserved motif (see Supplemetary Fig. 2 online) that is present in the other homologs and that we now know influences *K*_M_ and *k*_cat_, but not specificity.

^a^Enzyme names are associated with species as follows: ScCdc14, *Saccharomyces cerevisiae;* AnCdc14, *Aspergillus niger;* BcCdc14, *Botrytis cinerea;* CgCdc14, *Colletotrichum gloeosporioides;* CzCdc14, *Cercospora zeina;* MoCdc14, *Magnaporthe orzyae;* PsCdc14, *P. striiformis;* RsCdc14, *Rhizoctonia solani.*

**Figure 3:**
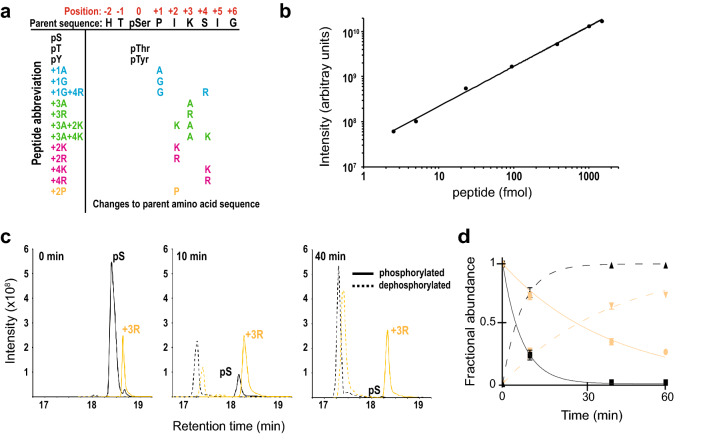
A novel assay for Cdc14 specificity profiling. (**a**) Nomenclature and sequences of the 15 synthetic phosphopeptides used to assess conservation of Cdc14 specificity in plant pathogenic fungi. The peptides are grouped by colors representing alterations to different determinants of ScCdc14 specificity: black, phosphoamino acid identity; cyan, + 1 Pro; green, + 3 Lys/Arg; magenta, additional basic amino acids around + 3. The orange peptide assessed the negative impact of Pro at + 2. (**b**) Linear dynamic range of the LTQ Orbitrap Velos Pro mass spectrometer for measuring phosphopeptide desphorphorylation by Cdc14. Mean integrated LC–MS peak intensities for the 15 phosphopeptides in (**a**) were plotted as a function of the injected amount of each peptide. For all specificity constant measurements, 750 fmol each peptide were injected. (**c**) Example of LC–MS chromatograms extracted with Skyline over a reaction time course with the synthetic phosphopeptide pool from (**a**). Signals for the phosphorylated and dephosphorylated forms of the pS and + 3R peptides are shown at 3 different reaction times. Integrated peak areas are used to calculated *k*_*cat*_/*K*_*M*_ as described in “Methods”. (**d**) Peak areas from data similar to (**c**) for the pS and + 3R peptides were used to calculate the fraction of substrate and product at different reaction times. Colors and lines match the peptide species from (**c**). Data were fit with a standard exponential reaction progress function, demonstrating that the MS assay accurately reflects expected enzyme reaction progress kinetics.

^b^The sequence for phosphopeptide substrate pS is in Fig. 3a.

^c^ND, not determined due to limited PsCdc14 enzyme.

Our primary interest was the conservation of substrate specificity. To define the specificities of the eight fungal pathogen Cdc14 homologs and compare them to the well-characterized ScCdc14, we developed a new assay that uses mass spectrometry (MS) to measure the specificity constant, *k*_*cat*_*/K*_*M*_, for a collection of synthetic phosphopeptide substrates (Fig. 3a). This assay, based on a similar assay for measuring protease specificity^38^, is more sensitive and has a much broader linear dynamic range (Fig. 3b) than the conventional malachite green colorimetric assay used to generate the phosphopeptide data in Table 1. These advantages made it convenient for measuring Cdc14 activity towards substrates whose specificity constants can vary by several orders of magnitude^32^. The assay also increases throughput by allowing simultaneous measurement of *k*_*cat*_*/K*_*M*_ for many different substrates. The peptides were based on the Yen1 Ser583 sequence with variations designed to test the importance of the four known ScCdc14 substrate recognition features (phosphoamino acid identity, + 1 Pro, + 3 Lys/Arg, Lys/Arg near + 3). Measurements were made by integrating MS chromatogram peak areas for the phosphorylated and dephosphorylated peptide species after Cdc14 treatment and using those values to calculate the fraction of substrate consumed (Fig. 3c; see “Methods” for details). A time course analysis demonstrated that the method accurately describes enzyme reaction progress (Fig. 3d), although a single time point is sufficient to calculate *k*_*cat*_*/K*_*M*_ for a given substrate^38^. Results from this assay were in reasonably good agreement with conventional steady-state kinetic measurements of *k*_*cat*_ and *K*_*M*_ using a malachite green spectroscopic assay (compare substrate pS in Tables 1 and 2), although the MS assay consistently yielded *k*_*cat*_*/K*_*M*_ ~ three to fivefold higher, likely due to sensitivity limitations of the malachite green assay with high affinity substrates.

**Table 2:** Specificity constant measurements for fungal pathogen Cdc14 homologs.

Peptide	ScCdc14	AnCdc14	BcCdc14	CgCdc14	CzCdc14	MoCdc14	RsCdc14	UmCdc14
pS	21,100 ± 1,400	25,200 ± 2,200	15,700 ± 1,200	19,400 ± 1,800	110,800 ± 10,600	17,000 ± 2,500	17,300 ± 1,500	12,900 ± 3,300
pT	3.9 ± 0.4	3.6 ± 0.1	9 ± 4	9 ± 4	13.7 ± 0.3	4 ± 1	2.8 ± 0.2	1.4 ± 0.1
pY	133 ± 2	110 ± 8	86 ± 10	87 ± 7	390 ± 21	121 ± 16	31 ± 2	24 ± 2
+ 1A	22 ± 5	30 ± 1	33 ± 3	22 ± 5	95 ± 16	25 ± 3	20 ± 2	11 ± 1
+ 1G	12 ± 1	116 ± 48	18 ± 3	16 ± 1	61 ± 9	13 ± 2	9 ± 2	4.7 ± 0.3
+ 1G+ 4R	30 ± 2	63 ± 1	64 ± 6	48 ± 6	171 ± 15	43 ± 4	21.4 ± 0.4	25 ± 7
+ 3A	116 ± 3	68 ± 13	38 ± 10	32 ± 3	171 ± 6	62 ± 5	64 ± 4	25 ± 1
+ 3R	4,110 ± 710	1,033 ± 52	546 ± 21	394 ± 41	3,000 ± 49	1,550 ± 71	499 ± 33	5,050 ± 170
+ 3A + 2K	93 ± 6	40 ± 4	370 ± 36	25 ± 4	89 ± 11	44 ± 7	33 ± 4	21 ± 1
+ 3A + 4K	260 ± 10	93 ± 19	69 ± 8	54 ± 4	262 ± 27	166 ± 10	62 ± 8	58 ± 3
+ 2K	14,800 ± 990	7,300 ± 2000	3,800 ± 980	10,000 ± 550	1,500 ± 490	8,700 ± 2,400	11,100 ± 1,200	ND
+ 2R	14,200 ± 720	12,600 ± 1,000	7,600 ± 710	12,400 ± 3,900	46,000 ± 4,000	11,000 ± 2000	4,600 ± 1,300	15,900 ± 9,100
+ 4K	42,400 ± 2,700	45,800 ± 2,500	27,900 ± 3,500	36,100 ± 2,700	79,200 ± 3,200	27,000 ± 6,400	14,100 ± 970	75,000 ± 1900
+ 4R	49,700 ± 2,400	49,400 ± 4,200	31,500 ± 880	37,100 ± 4,200	77,300 ± 430	30,700 ± 2,600	15,900 ± 2,100	73,700 ± 2000
+ 2P	218 ± 12	341 ± 13	179 ± 4	102 ± 25	1,510 ± 63	158 ± 18	132 ± 9	2,310 ± 210

All units are M^−1^ s^−1^. All values are the mean of 3 independent measurements ± standard deviations. ND, not determined.

**Figure 4:**
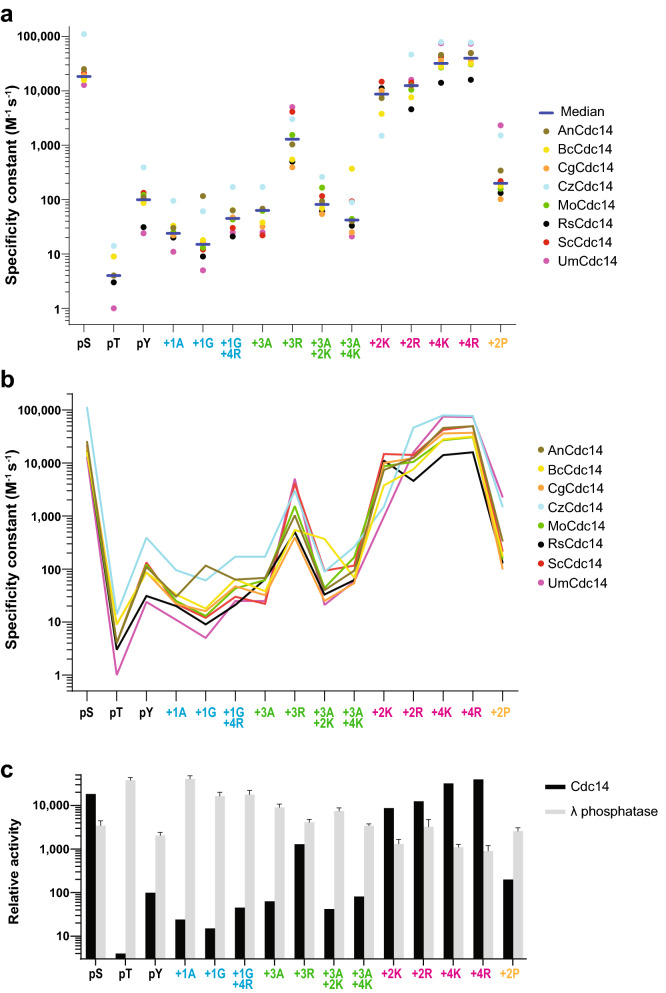
Substrate specificity is highly conserved in plant pathogenic fungal Cdc14 homologs. (**a**) The mean specificity constant, or *k*_*cat*_/*K*_*M*_, for the 8 indicated Cdc14 enzymes towards each of the 15 phosphopeptide substrates. The median value for each peptide is indicated with a short line. All data represent the average of 3 independent measurements. All *k*_*cat*_/*K*_*M*_ values with errors are reported in Table 2. Due to the limited concentration of purified PsCdc14 we could not complete its analysis under identical conditions. However, a specificity profile for PsCdc14 at a single enzyme concentration is shown in Supplementary Figure S4 online. (**b**) The data from (**a**) are shown in line graph format to better illustrate the degree of overlap in specificity. (**c**) To validate the full phosphopeptide collection and highlight the range of Cdc14 activities, the median Cdc14 specificity constants from (**a**) (units M^−1^ s^−1^) were compared to related values using the broad specificity lambda protein phosphatase. Since enzyme concentration was not available for the commercial lambda phosphatase, true *k*_*cat*_/*K*_*M*_ could not be calculated. Instead, lambda phosphatase values were normalized such that the maximal lambda and Cdc14 rates were equivalent.

Consistent with prior studies of ScCdc14, *k*_*cat*_*/K*_*M*_ values varied over several orders of magnitude for all fungal pathogen homologs (Fig. 4a and Table 2). Most importantly, all Cdc14 homologs exhibited very similar specificity profiles (Fig. 4a,b). These contrasted starkly with the profile of the broad specificity lambda protein phosphatase (Fig. 4c), which was used to validate the entire phosphopeptide collection and facilitate specificity constant calculations (see “Methods”). The similar specificity constants and profiles of all Cdc14 homologs clearly suggest that the three major determinants of ScCdc14 substrate specificity are invariant in plant fungal pathogen species within Dikarya. All homologs exhibited *k*_*cat*_*/K*_*M*_ values for the pSer-containing peptide > 1,000-fold higher than the pThr- and > 100-fold higher than the pTyr-containing peptides. Replacement of Pro at the + 1 position with Ala reduced *k*_*cat*_*/K*_*M*_ 477- to 1,168-fold (median 872). Similarly, replacement of Lys at + 3 with Ala reduced *k*_*cat*_*/K*_*M*_ 182- to 648-fold (median 464). Moving the single basic amino acid from the + 3 position to either + 2 or + 4 caused *k*_*cat*_*/K*_*M*_ reductions similar to the + 3 Ala substitution (median 567-fold and 249-fold, respectively), demonstrating that the + 3 position is critical in all enzymes.

In addition to these major determinants, other features of Cdc14 specificity were also conserved. All homologs exhibited a preference for Lys over Arg at the + 3 position, similar to ScCdc14, in many cases greater than tenfold. An additional Pro at the + 2 position, something we observed in a high throughput phosphopeptide library screen with ScCdc14 (unpublished observations), was a strong negative specificity feature, reducing *k*_*cat*_*/K*_*M*_ nearly 100-fold for all enzymes except UmCdc14. Prior studies showed that additional basic amino acids near the + 3 position could enhance ScCdc14 activity^33^. In the context of the Yen1 Ser583 sequence, however, these effects were minimal. An additional Lys or Arg at + 2 did not increase activity for any enzymes and an additional Lys or Arg at + 4 increased *k*_*cat*_*/K*_*M*_ only two to fivefold for several enzymes. We were unable to measure activity towards a peptide with 3 consecutive basic residues at + 3 to + 5 for technical reasons. The sequence around Yen1 Ser583 likely reflects a nearly optimal substrate for fungal Cdc14 enzymes. Collectively, the specificity profiling clearly demonstrates that Cdc14 enzymes from across the fungal kingdom possess a very strict, and highly conserved, substrate specificity that could make them susceptible to a common inhibitor structure.

### Cdc14 enzymes can be broadly, but specifically, inhibited by optimal substrate mimetics

**Figure 5:**
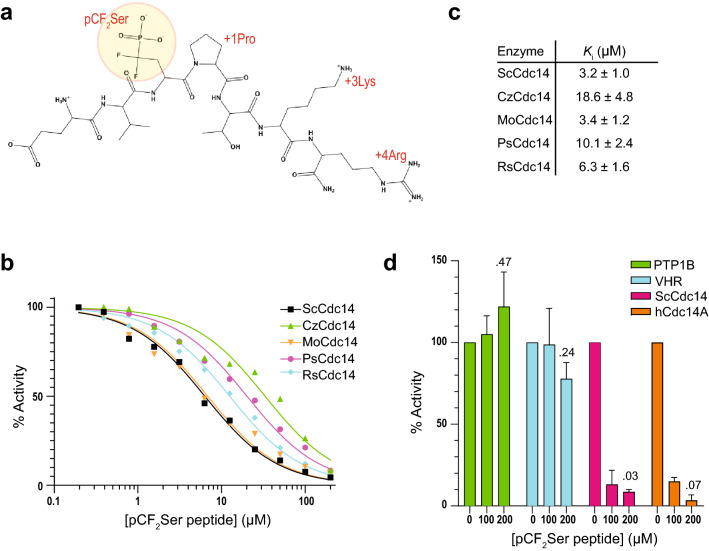
Fungal Cdc14 homologs can be specifically inhibited by a substrate mimetic. (**a**) Structure of the synthetic peptide used for inhibition assays (sequence Glu-Val-pCF_2_Ser-Pro-Thr-Lys-Arg-amide). (**b**) Dose response with the pCF_2_Ser peptide from A and the indicated fungal Cdc14 enzymes using DiFMUP as substrate. Data represent the average of 5 or 6 independent trials. It was not practical to show error bars on the graph, however error values for replicate *K*_*i*_ measurements are provided in (**c**). Best fit lines were generated with a standard slope dose response function with plateaus set at 100% and 0% in Graphpad Prism. We did not have enough pCF_2_Ser peptide to perform the full analysis on all 8 plant fungal pathogen homologs. (**c**) Each individual trial from the experiment in (**b**) was fit as described in b to generate IC_50_, from which *K*_*i*_ was calculated (see “Methods”). Values represent the mean ± standard deviation. (**d**) Comparison of pCF_2_Ser peptide inhibition of human tyrosine phosphatase PTP1B and dual specificity phosphatase VHR to ScCdc14 and human Cdc14A using DiFMUP at the measured *K*_*M*_ for each enzyme. Percent activity relative to a no inhibitor control was calculated and plotted. Data are means of 3 independent experiments and error bars are standard deviations. Numbers over the bars are p values from a *t-*test (unpaired, one-tail) comparing 0 and 200 µM inhibitor data.

The shared substrate specificity profiles suggested that optimal substrate mimetics could be the basis for effective broad-acting, but highly selective, inhibitors of fungal Cdc14 enzymes. To test this, we designed a peptide substrate mimetic containing a non-hydrolysable α,α-difluoromethlyene phosphonoserine (pCF_2_Ser) residue^39^ in place of pSer (Fig. 5a). The inhibitor peptide sequence Glu-Val-pCF_2_Ser-Pro-Thr-Lys-Arg is derived from a natural ScCdc14 substrate site in the Pds1/securin protein^40^ and contains the pSer, + 1 Pro, + 3 Lys and + 4 Arg features of an optimal substrate. The two fluorine atoms in the phosphonate α-methylene group of pCF_2_Ser are intended to match the electronegativity of a phosphate^39^, a strategy that has worked effectively for similar phosphonomethyl phenylalanine-based inhibitors of PTPs^41^.

The inhibitory constant (*K*_*i*_) for the pCF_2_Ser peptide against selected fungal Cdc14 homologs representing the full breadth of dikarya was determined from dose response assays using DiFMUP as a substrate at its *K*_*M*_ (Fig. 5b). Consistent with our hypothesis, Cdc14 homologs from diverse ascomycetes and basidiomycetes were inhibited similarly by this peptide, with *K*_*i*_ values ranging from 3 to 19 µM (Fig. 5c). We also tested inhibition of PTP1B and VHR, representing a well-studied classical nonreceptor PTP^42^ and a DSP related to Cdc14^26,43^, respectively, as well as human Cdc14A. Despite sharing similar active site architecture and catalytic mechanisms with Cdc14, PTP1B and VHR were not effectively inhibited at pCF_2_Ser peptide concentrations as high as 200 µM, (Fig. 5d). Human Cdc14A was inhibited similarly to ScCdc14. Although VHR activity was reduced to ~ 80% at 200 µM, this decrease was not statistically significant. Even if it were, it would equate to a *K*_*i*_ > 350 µM, roughly 2 orders of magnitude greater than that for the Cdc14 homologs. We conclude that substrate mimetic molecules should broadly inhibit fungal Cdc14 enzymes with minimal cross-reactivity towards other related phosphatases.

## Discussion

In this study we used a sensitive MS-based kinetic assay to simultaneously compare specificity constants for a collection of phosphopeptide substrates and showed that Cdc14 homologs from diverse fungal plant pathogens all recognize the same optimal substrate motif pSer-Pro-x-Lys with exquisite selectivity. We further provided proof of principle that mimicking the optimal substrate recognition motif may be a fruitful strategy for development of highly specific Cdc14 inhibitors. The MS assay will be useful in the future to define additional specificity determinants of Cdc14 enzymes using larger synthetic phosphopeptide pools or phosphopeptide pools derived from protease-treated cell extracts. For example, further exploration of the previously reported effect of multiple basic amino acids following pSer-Pro on Cdc14 activity^33^ is warranted, particularly in sequences with the sub-optimal Arg at the + 3 position. The assay should also prove useful in defining specificity of other phosphatases. It revealed previously unknown substrate preferences for lambda protein phosphatase, which incidentally tended to be opposite to those of Cdc14, including a > tenfold preference for pThr over pSer and pTyr, and a > tenfold decrease in specificity constant with the presence of a + 1 Pro.

Based on our results and prior studies we suggest that Cdc14 phosphatases have many attributes that make them an attractive target for antifungal development to combat fungal plant pathogens. First, as described in the introduction, Cdc14 appears important for plant infection by multiple fungal pathogens. In the future it will be important to characterize effects of CDC14 gene deletions in other fungal pathogen species to determine how extensively this requirement is shared. The molecular mechanisms by which Cdc14 promotes infection, including the identities of relevant substrates, are still undefined and should also be an active area of future research. Second, we have shown here that Cdc14 is completely absent from angiosperm plants, consistent with previous indications, making it less likely that specific inhibitors would adversely affect plant growth. Third, Cdc14 is nearly ubiquitous in the Ascomycota and Basidiomycota, the large fungal branches containing the majority of plant pathogens. Moreover, we have definitively demonstrated that the unusual specificity first characterized in ScCdc14^32,33^ is identical across the Ascomycota and Basidiomycota. This suggests that not only would Cdc14 be an attractive target, but inhibitors would likely have a broad spectrum of action against many pathogen species. Interestingly, genes encoding Cdc14 appear absent from the Glomeromycota that form arbuscular mycorrhizas in many plant species. Therefore, Cdc14-targeted antifungals would be predicted to have little or no negative impact on these beneficial plant symbionts, though this hypothesis should be directly tested. Fourth, the active site structure of ScCdc14 is known^27,44^, as is the structural basis for substrate recognition^27^. We demonstrated, in principle, that compounds successfully mimicking high affinity Cdc14 substrates can broadly, but selectively, inhibit fungal pathogen Cdc14 enzymes. Related to this, the Net1 protein in *S. cerevisiae* is a potent and selective competitive inhibitor of Cdc14 with a K_i_ of 3 nM^45^. Net1 could potentially serve as a useful template for design of specific small molecule Cdc14 inhibitors if high resolution detail of the structural basis for its inhibition of Cdc14 became available. A caveat is that Net1 is not conserved outside the Saccharomycotina, and therefore is absent from most plant pathogens, making it uncertain how broadly a mimetic compound would act. In any case, the existing high resolution structures of Cdc14-substrate complexes should be helpful in designing effective substrate-mimicking Cdc14 inhibitors that are biologically active.

There are important challenges that must be addressed if Cdc14 is to emerge as a useful antifungal target. Animal and fungal Cdc14 phosphatases are very similar. In fact, human Cdc14 can fulfill the essential Cdc14 function in *S. cerevisiae*^30^ even though it doesn’t perform a similar function in human cells^23^. Human Cdc14 enzymes also share similar specificity^32^, although they have not yet been characterized as thoroughly as ScCdc14, and we show here that specific inhibitors of fungal Cdc14 enzymes can similarly inhibit human Cdc14A. Thus, inhibitors targeted against fungal Cdc14 will likely be active against animal Cdc14 enzymes as well, raising the possibility of toxic side effects from consuming products from treated plants. However, the phenotypes reported for Cdc14 loss of function in animals with a single CDC14 gene suggest that side effects from collateral Cdc14 inhibition may be negligible. *C. elegans* and *D. melanogaster* develop normally and live full lives with very mild phenotypes in the absence of Cdc14 function^20,24^. In vertebrates, which typically have two widely expressed Cdc14 homologs, A and B, that exhibit distinct intracellular localization patterns and apparent functions^25^, full Cdc14 loss of function phenotypes have not yet been reported. However, individual loss of Cdc14B in mice and Cdc14A or B in cultured chicken, mouse and human cells has little to no effect on growth and development^19,21–23^. In contrast, strong Cdc14A or B repression by morpholino injection in zebrafish embryos leads to ciliogenesis defects and associated developmental abnormalities^46,47^. Thus, while existing data suggest that residual Cdc14-targeted antifungals in ingested plant products may not elicit significant side effects in humans and other animals, this issue must be experimentally addressed during any antifungal development. Future research to identify differences between fungal and animal Cdc14 enzymes that can be exploited for more specific antifungal development should also be a priority.

Another challenge in developing Cdc14-targeted antifungals will be the design of Cdc14 inhibitors that can effectively penetrate fungal cell wall and membranes to reach their target, which functions in the cytoplasm and nucleus. Phosphate compounds do not readily cross biological membranes and therefore peptide-based substrate mimetics like the one used in Fig. 5 that rely on phosphonate or other negatively charged phosphate isosteres may exhibit poor biological activity. Strategies to mask the negative charges on phosphate or phosphonate compounds exist and may be useful in overcoming this problem^48,49^. Another challenge is the potential difficulty identifying small molecules that potently and specifically inhibit Cdc14. The phosphate binding pocket of Cdc14, like other dual-specificity phosphatases^50^, is relatively shallow and high affinity inhibitors may be limited to relatively large compounds that interact with other Cdc14 substrate recognition sites, such as those for the + 1 Pro and + 3 Lys. In support of this, we have screened portions of several commercial small molecule libraries for Cdc14 inhibitors (roughly 50,000 compounds, our unpublished data). Only a few hits were observed, and most of them were compounds that likely react with the cysteine nucleophile in the Cdc14 active site. Taking advantage of the reactivity of the active site cysteine may be one strategy to pursue in the development of Cdc14 inhibitors^51^. Fragment based approaches to building larger inhibitors that can successfully mimic Cdc14 peptide substrates may be another useful strategy.

## Methods

### Phylogenetic analysis and bioinformatics

To identify protein sequences for phylogenetic analysis of Cdc14, we first queried the *Saccharomyces cerevisiae S288C* Cdc14 protein (YFR028C; ScCdc14) against NCBI RefSeq (release 97^52^) using phmmer (from the HMMER software package; https://hmmer.org). All sequences with a significant hit to YFR028C (global e-value ≤ 0.001) can be found in Supplementary Table S3 online. To be included in the final phylogenetic analysis, homologs to YFR028C needed to satisfy two criteria. First, the homolog must contain both Cdc14 domains, DSPc (PF00782) and DSPn (PF14671) as assessed by HMMER hmmscan (e-value ≤ 0.001). Second, the top hit in reciprocal best blastp searches of the homolog against the full human (Hg38) and yeast (*Saccharomyces cerevisiae 288C* R64) proteomes must be a Cdc14 sequence. Lastly, 11 sequences were excluded due to suspected contamination. The global and domain-level HMMER results, BLASTp hits/e-values, and potential notes regarding removal from the sample set can be found in Supplementary Table S3 online. Sequences that passed the filtering criteria above were aligned using MAFFT (–reorder –bl 30 –op 1.0 –maxiterate 1,000 –retree 1 –genafpair^53^), and alignment gaps were trimmed using TrimAl using the –gappyout parameter^54^. A maximum likelihood tree was constructed using iqtree (-alrt 1,000 -bb 1000^55^). Tree figures were generated using iTOL^56^. The full phylogeny and alignment can be found in Supplemental File S1.

We assessed patterns of conservation and loss of Cdc14 across plants, fungi, and animals by highlighting presence and absence patterns on a rough species tree. To get an estimate of the number of species represented by genome or transcriptome level data in RefSeq, a species had to be represented by at least 1,800 unique protein sequences to be included in the presence/absence analysis (Supplementary Table S4 online). Using the PhyloT web tool (phylot.biobyte.de; version 2019), we obtained the species tree of all plants, fungi, and animals in RefSeq in our custom database by providing their corresponding NCBI species taxonomy ids. The predicted status of Cdc14 homologs in each species’ genome was viewed on the species tree using iTOL^56^.

The multisequence alignment of all Cdc14 homologs used in this study was generated using default settings in Clustal Omega^57^ and processed in JalView^58^.

### Structural modeling

Molecular Operating Environment (MOE, Chemical Computing Group) was used to display sequence conservation on the ScCdc14 catalytic domain structure 5XW5^27^. The corresponding catalytic domain sequences of the eight ascomycete and basidiomycete homologs were globally aligned with the ScCdc14 sequence in MOE and the residues in the ScCdc14 structure colored by identity/similarity. MOE was also used to identify and display all ScCdc14 residues within 4.5 Å of the substrate peptide residues in 5XW5 that could contribute to substrate binding.

### Protein expression and purification

The coding sequences for the catalytic domains of fungal pathogen *CDC14* homologs were codon-optimized for *E. coli,* synthesized, and cloned into the *NdeI* and *BamHI* sites of pET15b for expression as N-terminal 6× histidine (6His) fusion proteins by Genscript Corp. Figure S2 provides the exact expressed protein sequence for each homolog. To enhance solubility, coding sequences for the non-conserved and variable length C-terminal regions following the catalytic domains were omitted. 6His-Cdc14 enzymes were expressed in 1 L cultures of BL21 AI cells (Thermo Fisher Scientific) by induction with 0.02% l-arabinose overnight at 25 °C. Cells were lysed with 1 mg/mL lysozyme for 30 min on ice in 30 mL 25 mM HEPES pH 7.5, 500 mM NaCl, 10 mM imidazole, 0.1% Triton X-100, 10% glycerol, 1 mM PMSF, 10 µM leupeptin, 1 µM pepstatin, and 1,000 units Universal Nuclease (Thermo Fisher Scientific). Extracts were clarified by centrifugation at 35,000×*g* for 30 min and the soluble fraction was loaded on a 1 mL HisTrap column (GE Healthcare) equilibrated with 25 mM HEPES pH 7.5, 500 mM NaCl, and 10% glycerol. The column was washed at 10 mM and 25 mM imidazole prior to elution with a gradient from 25 to 250 mM imidazole. Peak fractions were pooled and dialyzed overnight into 25 mM HEPES pH 7.5, 300 mM NaCl, 2 mM EDTA, 0.1% 2-mercaptoethanol, and 40% glycerol and stored at − 80 °C in small aliquots. GST-hCdc14A was purified exactly as described^31^. Protein concentrations were determined using a spectroscopic dye assay (Bio-Rad Laboratories) and bovine serum albumin as a standard.

### Steady-state enzyme kinetics

#### pNPP assay

Activities towards varying concentrations of *para*-nitrophenyl phosphate (pNPP) were measured in 100 µL assay buffer (25 mM HEPES pH 7.5, 2 mM TCEP, 1 mM EDTA, and 150 mM NaCl) at 30 °C. Enzyme concentrations were chosen to achieve absorbance values within the linear response range while satisfying the steady-state assumption where substrate consumption was < 1%. Reactions were initiated by enzyme addition and stopped with 1 N NaOH. Absorbance at 405 nm was measured on a Synergy H1 microplate reader (BioTek) and converted to product concentration using a *para*-nitrophenol standard curve. Initial rates were plotted as a function of substrate concentration and fit with the Michealis-Menten equation in GraphPad Prism (Version 8) to determine *k*_*cat*_ and *K*_*M*_.

#### Phosphopeptide assay

Activities towards varying concentrations of the synthetic phosphopeptide HT(pSer)PIKSIG (Genscript Corp) were measured in 50 µL assay buffer at 30 °C essentially as described above for pNPP, except substrate consumption was limited to 10% due to limited assay sensitivity and the reaction was stopped with 100 µL Biomol Green (Enzo Life Sciences). Absorbance was measured at 640 nm and converted to product with a sodium phosphate standard curve. *k*_*cat*_ and *K*_*M*_ were calculated as described above.

#### DiFMUP assay

Activities towards varying concentrations of 6,8-difluoro-4-methylumbelliferyl phosphate (DiFMUP, Thermo Fisher Scientific) were performed under the same conditions described above for pNPP with the exception of VHR, which was assayed in 50 mM Bis–Tris (pH 6.0), 1 mM DTT, and 100 mM NaCl. All enzyme concentrations were 0.5 nM, except for PsCdc14 and VHR, which were assayed at 2 and 5 nM, respectively. Fluorescence intensity was measured continuously on a Synergy H1 microplate reader (BioTek) with excitation and emission wavelengths set at 358 and 450 nm, respectively. Fluorescence intensity was converted to product concentration using a 6,8-difluoro-4-methylumbelliferone standard curve. Background fluorescence was subtracted from each reaction and initial rates were calculated from the slope of the linear portion of the product concentration versus time plots. *k*_*cat*_ and *K*_*M*_ were calculated as described above.

### Specificity constant (*k*_*cat*_/*K*_*M*_) measurements

#### Enzyme reactions

*k*_*cat*_/*K*_*M*_ measurements can be made on pooled substrate mixtures for characterizing enzymatic specificity, provided substrate concentrations are far below *K*_*M*_^38^. Mass spectrometry is a useful tool for monitoring reaction progress in such mixtures, as long as each substrate and product has a unique mass. Phosphopeptides with unique sequences and masses (Fig. 3a) were synthesized and purified by Genscript Corp. *K*_*M*_ for optimal ScCdc14 substrate peptides are typically 50–100 µM^32^. Therefore, reactions contained 375 nM each synthetic phosphopeptide and were initiated by addition of Cdc14 (concentrations ranged from 1 to 1,000 nM) in 200 µL assay buffer at 30 °C. 60 µL aliquots were removed and mixed with 60 µL 5% formic acid at desired times to stop the reactions. Peptides were desalted on C18 spin columns (Thermo Fisher Scientific) and dried by vacuum centrifugation prior to MS analysis.

#### Liquid chromatography (LC)/MS analysis

MS analyses were conducted with an LTQ Orbitrap Velos Pro mass spectrometer coupled to an EASY-nanoLC 1,000 chromatography system (Thermo Fisher Scientific). Peptides were separated on a homemade column (45 cm × 360 µm OD × 75 µm ID) packed with ProntoPEARL C18 resin (2.2 µm particle size, 100 Å pore size; Bischofff Chromatography) at a flow rate of 250 nL/min and directly injected into the Velos. Peptides were loaded in 3% acetonitrile/0.1% formic acid and eluted with a multistep gradient of increasing acetonitrile: 3% to 35% over 10 min, 35% to 50% over 5 min, and 50% to 90% over 5 min. The Velos was operated in data dependent acquisition mode with cycles of one MS survey scan (200 to 1,100 m/z, 60,000 Hz resolution at 400 m/z) followed by 10 MSMS scans (normalized collision energy − 35%, 12 s dynamic exclusion). Raw data were searched with MaxQuant (https://www.maxquant.org) against a custom database containing a background of all *S. cerevisiae* proteins and all synthetic peptide sequences. The MSMS results were used to build a spectral library in Skyline (https://www.skyline.ms), which then allowed identification and integration of confirmed LC chromatogram peaks for each phosphorylated and dephosphorylated peptide. R and Excel were used to calculate ionization correction factors for each phosphorylated/dephosphorylated peptide pair and the specificity constant, *k*_*cat*_/*K*_*M*_*,* from the integrated LC peak values.

#### Specificity constant calculations

As described previously^38^, at substrate concentrations well below *K*_*M*_, free enzyme is approximately equal to total enzyme and substrate competition is negligible. Under these conditions, formation of enzyme–substrate complex becomes rate limiting, and observed reaction progress for each substrate can be related to the second order rate constant *k*_*cat*_/*K*_*M*_ via the following equation (based on^38^):

1 $${S}_{t}={e}^{-\frac{{k}_{cat}}{{K}_{m}}{E}_{0}t}$$

where *S*_*t*_ is the fraction of substrate remaining at time t and E_0_ is the total molar enzyme concentration. We used a previously published strategy for label-free quantification of the stoichiometry of peptide modifications^59^ to directly calculate *S*_*t*_ from the LC peak areas within a given sample, eliminating the need to normalize between different timepoints or use isotope labeling. This required calculation of a unique ionization correction factor for each substrate/product pair so that their integrated signals could be directly compared. Ionization correction values were determined by treating the phosphopeptide pool with lambda protein phosphatase (New England Biolabs) to generate different substrate:product ratios. Once *S*_*t*_ was calculated, Eq. (1) was rearranged to solve for *k*_*cat*_/*K*_*M*_.

### pCF_2_Ser synthesis and peptide incorporation

The non-hydrolyzable phosphonate analog of phosphoserine, (α,α-difluoromethylene)phosphonoserine (pCF_2_Ser) was synthesized using a hybrid scheme based on two prior reports^49,60^ that are reviewed in^39^. A full description of the synthesis is provided in Supplementary Material online.

pCF_2_Ser was incorporated into the peptide sequence Glu-Val- pCF_2_Ser-Pro-Thr-Lys-Arg by Fmoc-solid phase synthesis on Chemmatrix Rink Amide resin (100 mg, 0.22 mmol/g). The desired amino acids were sequentially coupled to the free amine using HATU (4 eq, 0.09 mmol) and diisopropylethylamine (8 eq, 0.15 mmol) as coupling agents dissolved in DMF. Deprotection of all amino acid side chains, including the ethyl protecting groups on pCF2Ser, and simultaneous cleavage of peptide from the resin were carried out as described previously^61^. Crude peptide was dried, dissolved in 10% aqueous acetic acid and washed with chloroform to remove non-volatile by-products. The peptide was purified to homogeneity using reverse phase HPLC and confirmed by matrix-assisted laser desorption ionization time-of-flight MS.

### Enzyme inhibition

Inhibition of fungal Cdc14 enzymes by pCF_2_Ser peptide was measured using the DiFMUP assay described above. DiFMUP concentration was set at the measured *K*_*M*_ value for each enzyme and pCF_2_Ser peptide concentration was varied. Measured initial rates were converted to percent activity relative to a reaction lacking pCF_2_Ser peptide. Percent activities were plotted as a function of pCF_2_Ser peptide concentration and fitted with a standard dose–response function with plateaus set at 100% and 0% to determine IC_50_ in GraphPad Prism (Version 8). *K*_*i*_ is related to IC_50_ by the equation *K*_*i*_ = IC_50_/([S]/*K*_*M*_ + 1). Since [S] was equal to *K*_*M*_ in our assays, *K*_*i*_ = IC_50_/2.

## Supplementary information

Supplementary Information 1.

Supplementary Information 2.

Supplementary Tables.
