# Supplementary material for: Conservation of Cdc14 phosphatase specificity in plant fungal pathogens: implications for antifungal development
Source: Sci Rep. 2020 Jul 21;10:12073. doi: 10.1038/s41598-020-68921-3 (PMC7374715; doi:10.1038/s41598-020-68921-3)
Supplement: Supplementary file 2 — Supplementary Information 2. [file 41598_2020_68921_MOESM2_ESM.pdf]

SUPPLEMENTARY MATERIAL

Conservation of Cdc14 phosphatase specificity in plant fungal pathogens: implications for antifungal development

Andrew G. DeMarco[1], Kedric L. Milholland[1], Amanda L. Pendleton[1,4], John J. Whitney[1], Peipei Zhu[1,2], Daniel T. Wesenberg[1], Monessha Nambiar[2], Antonella Pepe[3,†], Stefan Paula[1,2,‡], Jean Chmielewski[2,3], Jennifer H. Wisecaver[1,4], W. Andy Tao[1,2,3], Mark C. Hall[1,3]

[1]Department of Biochemistry, [2]Department of Chemistry, [3]Center for Cancer Research, and [4]Center for Plant Biology, Purdue University, West Lafayette, IN 47907

[†]Current Address: Institute of Chemical Biology and Drug Discovery, Stony Brook University, Stony Brook, NY, 11794-3400

[‡]Current Address: Department of Chemistry, California State University, Sacramento, 6000 J Street, Sacramento, CA 95819

Contents:

Supplementary Methods

Supplementary Figures S1-S4 Legends

Supplementary Figures S1-S4

SUPPLEMENTARY METHODS

pCF2Ser synthesis.

pCF2Ser was prepared as shown in Scheme 1 and following synthetic protocols published by Berkowitz *et al.* [59] and Arrendale *et al.* [49].

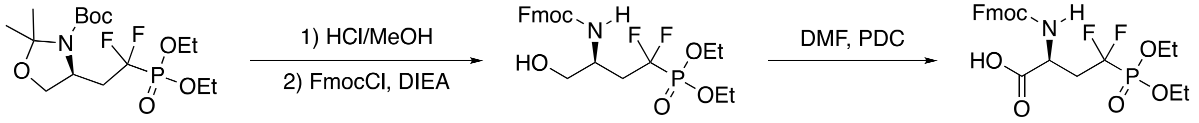

1 2 3

Scheme 1:Synthesis of non-hydrolyzable phosphonate analog of phosphoserine

General procedure

tert-Butyl (S)-4-(2-(diethoxyphosphoryl)-2,2-difluoroethyl)-2,2-dimethyloxazolidine-3-carboxylate (**1**) was prepared as reported by Berkowitz *et al.* [49]. All reagents were used as obtained from commercial sources and used directly. ^{1}H and ^{13}C NMR spectra were recorded on Bruker 500 MHz Avance III HD spectrometer. ^{1}H chemical shifts values (δ) are referenced to the residual nondeuterated components of the NMR solvents (δ = 7.26 ppm for $CHCl_3$ and δ = 2.50 ppm for DMSO-d_5. The ^{13}C chemical shifts (δ) are referenced to $CDCl_3$ (central peak, δ = 77.0 ppm), DMSO-d6 (central peak, δ = 39.5 ppm). Signals are listed in ppm, and multiplicity identified as s = singlet, br = broad, d = doublet, t = triplet, q = quartet, m = multiplet. Automated flash chromatography was performed on Yamazen W-Prep 2XY using Yamazen Universal Premium silica gel (30 mm 60 Å) columns. Analytical thin-layer chromatography (TLC) was carried out on silica gel 60 F254 aluminum plates and eluted plates were visualized with nihydrin stain. Mass spectral data were obtained from the Purdue University Metabolite Profiling Facility, West Lafayette, IN on an Agilent 6550 QTOF mass spectrometer coupled to an Agilent 1200 series high-performance liquid chromatography system.

Synthesis of (9*H*-fluoren-9-yl)methyl (*S*)-(4-(diethoxyphosphoryl)-4,4-difluoro-1-hydroxybutan-2-yl)carbamate (2) [59]

tert-Butyl (*S*)-4-(2-(diethoxyphosphoryl)-2,2-difluoroethyl)-2,2-dimethyloxazolidine-3-carboxylate (**1**) [59] (1.35 g, 3.37 mmol) was dissolved in anhydrous MeOH (30 mL) and the resulting clear solution was cooled in ice/water bath. Conc. HCl (3.00 mL) was added dropwise with vigorous stirring and then the cold bath was removed and the reaction mixture was stirred at room temperature for 2 hours. Upon disappearance of the starting material, the solvent was evaporated under reduced pressure and water was stripped by azeotropic evaporation with CH_3CN (2x 30 mL) and toluene (1x 30 mL). Dichloromethane (45 mL) was added to the crude mixture, and the solution was cooled in ice/water bath. Then *N*,*N*-diisopropylethylamine (1.76 mL, 10.1 mmol) and fluorenylmethyloxycarbonyl chloride (945 mg, 3.71 mmol) were added. The reaction mixture was stirred for 18 h with slow warming up to room temperature and then it was quenched with a saturated aqueous solution of $NaHCO_3$ (75 mL). The aqueous layer was extracted with CH_2Cl_2 (3 x 90 mL) and the combined organic layers

were washed with brine and dried over anhydrous Na_2SO_4. The solvent was evaporated and the crude material was purified by automated flash chromatography using a 55 g silica gel column, and a mixture of ethyl acetate in hexanes with a gradient of 20-70% (R_f = 0.3, 100% ethyl acetate). The title compound was isolated as a foaming colorless solid in 51% yield (unoptimized yield, 840 mg).

^{1}H NMR (500 MHz, Chloroform-*d*) δ 7.77 (ddd, *J* = 7.5, 0.89, 0.89 Hz, 2H), 7.60 (ddd, *J* = 7.5, 2.1, 1.1 Hz, 2H), 7.40 (ddd, *J* = 7.5, 7.5, 0.89 Hz, 2H), 7.32 (ddd, *J* = 7.5, 7.5, 1.1 Hz, 2H), 5.38 (d, *J* = 7.7 Hz, 1H), 4.40 (d, *J* = 7.1 Hz, 2H), 4.35 – 4.18 (m, 5H), 4.07 (brs, 1H), 3.77 (bs, 2H), 2.44 (m, 3H), 1.38 (td, *J* = 7.1, 2.1 Hz, 6H). ^{13}C NMR (126 MHz, $CDCl_3$) δ 156.1, 143.8, 141.3, 127.7, 127.1, 125.1, 123.5 – 116.7 (m, CF_2), 120.0, 66.9, 64.9 (t, *J* = 6.2 Hz), 64.7, 47.9, 47.2, 35.0 (m), 16.4, 16.37. ^{31}P NMR (203 MHz, Chloroform-*d*) δ 6.48 (t, *J* = 106.4 Hz). Spectral data are in agreement with those reported.

Synthesis of (*S*)-2-((((9*H*-fluoren-9-yl)methoxy)carbonyl)amino)-4-(diethoxyphosphoryl)-4,4-difluorobutanoic acid (3) [49]

Pyridinium dichromate (3.92g, 6.00 equiv.) was added to a colorless, solution of **(2)** (840 mg, 1.74 mmol) in DMF (8.00 mL) and the bright orange suspension was stirred overnight. After 16 h, the reaction mixture appeared as a dark brown suspension and it was diluted with water (70 mL) and ethyl acetate (100 mL). The aqueous layer was separated and extracted with ethyl acetate (3 x70 mL). The combined organic layers were washed with brine and dried over anhydrous sodium sulfate. The solvent was removed under reduced pressure and the resulting yellow oil was purified by flash column chromatography on silica gel. Ethyl acetate in hexanes was used as eluant with a gradient of 10-100% to elute the less polar fractions and then methanol in ethyl acetate with a gradient of 1-5% was used to elute **3**. The title compound was isolated as a white solid in 33% yield (unoptimized yield) [49, 60] (291 mg). The optical purity of **3** was assessed by measuring its specific optical rotation, which was slightly higher than the value reported for the same compound. [49] In addition, **3** was reacted with (*S*)-α-methylbenzylamine and separately with racemic α-methylbenzylamine. Comparing ^{1}H and ^{19}F NMR of the resulting amides it was possible to confirm that **3** was essentially optically pure.

$[\alpha]^{23}_D$= + 5.6 (c = 1.15, $CHCl_3$), lit. $[\alpha]^{25}_D$= + 4.3 (c = 0.923, $CHCl_3$). [49]

^{1}H NMR (500 MHz, DMSO-d_6, 65 °C) δ 7.86 (ddd, *J* = 7.5, 1.0 Hz, 2H), 7.68 (d, *J* = 7.5 Hz, 2H), 7.40 (dd, *J* = 7.5, 7.5 Hz, 2H), 7.30 (ddd, *J* = 7.4, 2.0, 1.0 Hz, 2H), 4.41 – 4.10 (m, 7H), 3.43 – 2.89 (m, 3H), 2.64 – 2.40 (m, 1H), 1.29 (t, *J* = 7.0 Hz, 6H). ^{13}C NMR (126 MHz, DMSO, 65 °C) δ 172.9, 156.0, 144.3, 141.2, 128.0, 127.5, 125.6, 121.6 (d, *J* = 47.7 Hz) 120.5, 66.2, 64.7, 48.9, 47.3, 35.6, 16.63, 16.59. ^{19}F NMR (471 MHz, DMSO-d_6, 65 °C) δ -111.98 (ddd, *J* = 293.4, 104.3, 26.1 Hz), -113.29 (dddd, *J* = 297.4, 106.6, 28.7, 10.0 Hz). ^{31}P NMR (203 MHz, DMSO-d_6) δ 6.39 (t, *J* = 103.3 Hz). HRMS (ESI), $[M+Na]^+$ calculated for $C_{23}H_{26}F_2N_1O_7PNa$ 520.1307, found 520.1311. Spectral and analytical data are in agreement with those reported.

SUPPLEMENTARY FIGURE LEGENDS

Figure S1. Maximum likelihood phylogeny of Cdc14 sequences. Branches are color coded to indicate their taxonomic association. The size of the grey circles on branches indicate the strength of the SH-like support values; the larger the circle, the greater the support. If no circle is present, the branch has > 0.95 support.

Figure S2. Alignment of Cdc14 homologs from plant fungal pathogens. The expressed sequence of ScCdc14 and Cdc14 homologs from 8 plant pathogenic fungi (initially identified by BLAST using ScCdc14 as query) representing diverse ascomycete and basidiomycete lineages were aligned in Clustal Omega using default settings. The aligned sequences reflect the truncated coding sequences included in the pET15b expression constructs for purification from *E. coli*, with the exception of the sequence from *U. maydis.* The *CDC14* gene from *U. maydis* has an unusual 5' extension, which was also truncated, and the 3' tail following the catalytic domain was truncated at a different site. The truncation sites for UmCdc14 are indicated by the gray highlighting. The common truncation site for the other homologs was chosen based on unpublished data from our lab indicating that all sequence following the conserved QPRK motif at residues 434-437 of ScCdc14 are dispensable for full catalytic activity. Cyan highlighting indicates amino acids contributing to substrate recognition around the Cdc14 active site, as in Figure 2d.

Figure S3. Purification of plant pathogenic fungal Cdc14 homologs. SDS-PAGE analysis, with Coomassie blue staining, of identical amounts of recombinant plant pathogen Cdc14 homolog catalytic domains purified from E. coli by nickel affinity chromatography.

Figure S4. Substrate specificity profile of PsCdc14. Data are equivalent to those for the other homologs in Figure 5A. However, PsCdc14 was recovered at relatively low concentration, apparently due to limited solubility. As a result, we were unable to perform assays at all the same enzyme concentrations used for the other homologs. This prevented the generation of suitable substrate:product ratios for accurate calculation of k_{cat}/K_M for some of the peptides. However, the relative reaction rates are still appropriate for comparison to each other to establish the PdCdc14 specificity profile, which is clearly similar to the other Cdc14 homologs. Data points are the average rates from 3 independent experiments at a single enzyme concentration and error bars are standard deviations.

Figure S1

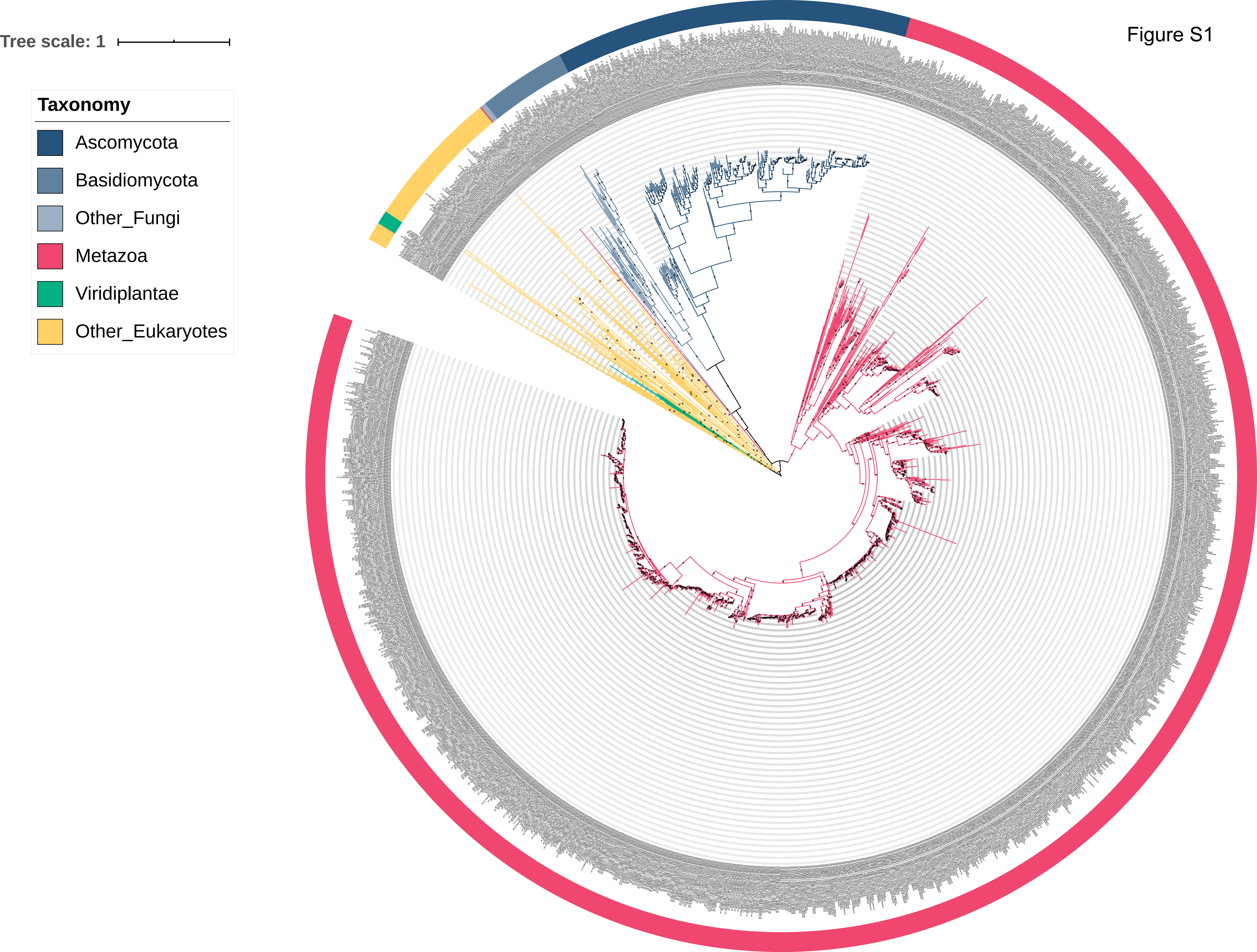
Tree scale: 1
Taxonomy
Ascomycota
Basidiomycota
Other_Fungi
Metazoa
Viridiplantae
Other_Eukaryotes

Figure S2

```
S.cerevisiae            ------------------------------------------------------------       0
C.zeina                 ------------------------------------------------------MPHQSR       6
A.niger                 ------------------------------------------------------------       0
B.cinerea               ------------------------------------------------------------       0
M.oryzae                ------------------------------------------------------------       0
C.gloeosporioides       ------------------------------------------------------------       0
U.maydis                MASTSAAFVLQDRQDHIIAPDDSDFLDSDAESSSTPHPDDQLIEEAVLQGFENASPQRSS       60
R.solani                ------------------------------------------------------------       0
P.striiformis           -------------------------METTQQDSQ-NQQIDCLMADV--------STKRLN       26

S.cerevisiae            --------MRRSVYLDNTIEFLRGRVYLGAYDYTPED----------------------- 29
C.zeina                 S-S--VQEASAMTSFGQVIEYIQDRLYLASYTHTPDESTPFPYPAKQS--RSPHKSRSSR 61
A.niger                 --------MATNSGYGQVIEYIQDRLYLASYDNPPDARTPFPYPLEQ--PRSPSK-RSAR 49
B.cinerea               --M--P--PSSQLKYGQIIEYIPDRLYLASYTTPPSPDTLFPYPEQQSSRRSPTK-RSAR 53
M.oryzae                --M--APSISKSSNMGQVIEYIENRLYLASYVNPPEADTLFPYPEPP--TRSPTK-RSRA 53
C.gloeosporioides       --------------MGQVIEYIPDRLYLAAYIHPPTAETIFPYGEPV--QTSPRK-RSQR 43
U.maydis                R-NAAGATSANKLWPAHPLIRIHKRLWFTWFNGDLPSEDDLNEQDG----LPPAW-KNFD 114
R.solani                -----------MAPVCEPLCRFSDRLYWTSFPAPAPSPDQLNRPEQ----PAPAL-RTKA 44
P.striiformis           LPIQPIASSSAIRTPSSPISQFGPRLGLACFSNPIPDPLTLNSTHG-------------- 72
                                          :  :  *:    :
S.cerevisiae            --------------TDELVFFTVEDAIFYNSFHLDFGPMNIGHLYRFAVIFHEILNDPEN 75
C.zeina                 AQAGPQSA--AAAPQHPPFYFSIDHTLLYNAFHADFGPLHIGHLYRFAVQLHEVLGHPDN 119
A.niger                 AQPS------TPSKKRSPVYFTVDDVLLYNSFHADFGPLHIGHLYRFAVQFHEILGEPEN 103
B.cinerea               AADGPSVV---SDKREPPHYFSVDDTLLYNAFHHDFGPLHIGHLYRFAVQFHDILGAPEN 110
M.oryzae                AV-EPTP----VAARKQPYYFSIDDSLLYNAFHHDFGPLHIGHLYRFALEFHDILGAKEN 108
C.gloeosporioides       AAEGPTPV-QSAAVRPTPYYFSVDDTLLYNAFHHDFGPLHIGHLYRFALQFHDVLGAKEN 102
U.maydis                DAEVKKYIDPTETNPSRLHWFNIDHDLVYLSFWFDWGPLNVGMFYRFCLHVHHMLTDPQM 174
R.solani                RAAGPTPVSP--EENHLYYYFCIDDRLLYLSFFEDWGPLNLAQVYRACILIHELLQDANL 102
P.striiformis           -------FIQ--SNLQPVYWFTIDSLLIYLSFYQDTGPLNCACLYRFCLHLHELLEDSSM 123
                                           :* ::  :.* :*  * **:: . .** .: .*.:*    .
S.cerevisiae            ANKAVVFYSSASTRQRANAACMLCCYMILVQAWTPHQVLQPLAQVDPPFMPFRDAGYSNA 135
C.zeina                 ENRPVVFWSHADSRSRANAACILATYMVLIQNWPPHLALAPIAQMDPPCMPFRDAGYSQA 179
A.niger                 RDRAVVFYSKTDARSRANAACLVACYMVLIQAWPPHLALAPIAQADPPYMPFRDAGYSQA 163
B.cinerea               KNKPVVFWSKADARSRANAACLLATYMVLIQSWAPHLALAPIAQADPPLMPFRDAGYSQA 170
M.oryzae                KDRPVVFWSRADPRSRANAACVLACYMVLIQSWPPHLALAPIAQVDPPLMPFRDAGYSTA 168
C.gloeosporioides       KDRPIVFWSRADPRSRANAACMLACYMVLIQNWPPHLALAPIAQVDPPLMPFRDAGYSQA 162
U.maydis                HDATLVLYTTNAPAQKANAALLVTLYAMIIGKVTPADAFYPISEL--EFKPFRDAGYGRA 232
R.solani                KRHRIVLYSSSDPRRRANSALLMALYCMIVQRRTPWEVFYPIAEV--EFMPFRDAGRGRS 160
P.striiformis           SDRRIILYSSDDPDKKANAALLMALYCMIVLRWSVADALHPISHL--ELQPFRDAGYSRA 181
                            :::::      :**:* ::  * :::       .: *::.      ****** . :
S.cerevisiae            DFEITIQDVVYGVWRAKEKGLIDLHSFNLESYEKYEHVEFGDFNVLTPDFIAFASPQEDH 195
C.zeina                 DYSLTIQDVVYGVWKAKEENLVGLKEFSLEEYEKYERVDMGDFNWVSPDFLAFASPQHLP 239
A.niger                 DFILNIQDVVYGVWKAKEQSLCSLRDFSLEEYEKFERVDMGDFNWITPQFLAFASPQHQP 223
B.cinerea               DYGITVQDVVYGVWRAKEQGFCALPQFDLEEYERYERVDQGDFNWLTPDFLAFASPQHHP 230
M.oryzae                DYGITVQDVVYGVWKAKEEGCVMLDSFDLGEYEKYERVENGDFNWITPHFLAFASPMHYP 228
C.gloeosporioides       DYGITVQDVVYGVWKAKEEGCCALDNFDLDEYEKFERVEHGDFNWITPHFLAFASPQHTP 222
U.maydis                DYNLSIQDILYGVHRAINSGLLDLTTFNLDEYEKYEQVSHGDWNWITPNFIAFASPNDRN 292
R.solani                DFHLGIQDCLWGVWKAQHNHLLDMNEFSVDEYEYYEKVDNGDWNWITPGFIAFASPVDSV 220
P.striiformis           DFNLSIQNVLFGVKKAIDLRLLKLEEFDLKEYETFEKVEHGDYNWLSPHFIAFASPVESA 241
                        *: : :*: ::** :* .     :  *.: .** :*:*. **:* ::* *:***** .
S.cerevisiae            PKGYLAT----------------KSSHLNQPFKSVLNFFANNNVQLVVRLNSHLYNKKHF       239
C.zeina                 THVFQPT-EPLYAQLPRTVSAVKRDSNLPTPFKNVLTHFAERGIGLVVRLNSELYSPSYF       298
A.niger                 IAPIPQNT-PEFAALPSTVSEVL-SSKLPLPFKNVLAHFSSRNIGLVVRLNSELYSPSYF       281
B.cinerea               VQPILPSS-PLYASLPTTLEDVDTHPTLPTPFKNVLKHFTSRNIGLVVRLNSELYSPSFF       289
M.oryzae                VDRVTETMGEAYKLLPKTIADVNKHPDLPDPFKNVLCHFSEKNIGLVVRLNSQLYSPSYF       288
C.gloeosporioides       VAKVIEGT-EEWDELPKSIGQVDANKALPQPFKNVLKHFTERNIGLVVRLNSPLYSPSFF       281
U.maydis                YVEALRVG---EGRLPAS--YSFRPPGEDKLFGKTIRYFKDRGVKLVVRLNNPLYDREAF       347
R.solani                WIRQQKEG---KTKGGAS----ALQRKLPTPFMNVLEYFSERNVKLVVRLNNELYDKAVF       273
P.striiformis           NG------------------------RIGKAFKMILDQFDRVNVKLVIRLNKKLYDETRF       277
                                                       *   :  *   .: **:***. **.   *

S.cerevisiae            EDIGIQHLDLIFEDGTCPDLSIVKNFVGAAETI-IKRGGKIAVHCKAGLGRTGCLIGAHL 298
C.zeina                 TALGIKHLDMIFDDGTCPPLNLVKKFISLAHQMINEKRKGIAVHCKAGLGRTGCLIGAYL 358
A.niger                 TALGITHVDMIFEDGTCPPLPLVRRFIKMAHETIS-KKKGIAVHCKAGLGRTGCLIGAYL 340
B.cinerea               TALGIEHLDMIFDDGTCPPLSVVRKFITLAHEMITVQKRGIAVHCKAGLGRTGCLIGAYL 349
M.oryzae                ESLGIRHLDMIFEDGTCPPLSLVRKFIRLAHETITIRKRGIAVHCKAGLGRTGCLIGAYL 348
C.gloeosporioides       ESMGISHLDMIFDDGTCPPLTTVRKFIRLAHETITVKKKGIAVHCKAGLGRTGCLIGAYL 341
U.maydis                LNVGIDHSDMYFDDGSNPTDEILADFIAKADHVI-AHDGVVAVHCKAGLGRTGVLIGAYL 406
R.solani                EERGIEHLDLYFDDGTNPADDITRTFIAKSDAII-EAGGAIAVHCKAGLGRTGTLIGAYL 332
P.striiformis           TKRGIAHREMYFDDGTNPTMEMVREFITISERII-GAGGVVAVHCKAGLGRTGTLIGAYL 336
                           ** * :: *:**: *       *:  :.         :************ ****:*
```

```
S.cerevisiae          IYTYGFTANECIGFLRFIRPGMVVGPQQHWLYLHQNDFREWKYTTRISLKPSEAIGGLYP    330
C.zeina               IYRYGFTANEVIAFMRFMRPGMVVGPQQHWLHLNQGTFREWWFEDTMREKLMASMQPATP    390
A.niger               IYRYGFTANEIIAFMRFMRPGMVVGPQQHWLHLNQGSFREWWYEDSMKEKLAQMQAA---    372
B.cinerea             IYRYGFTANEIIAYMRFMRPGMVVGPQQHWLHLNQGTFREWWIEEQFEIRMKEKLANMAP    381
M.oryzae              IYRHGFTADEVISYMRFMRPGMVVGPQQHWLHLNQGVFREWWVEERVARKLRKELAAAAQ    380
C.gloeosporioides     IYRHGFTANEIIAFMRFMRPGMVVGPQQHWLHINQGIFREWWIEERIERKLRKEMAA---    373
U.maydis              VWKHGFSAGEAIGFMRFMRPGCVVGPQQHFMYQNFAEWIKWGVRDHAMKEARQLIAEERT    438
R.solani              IYKYGFTASEAIGFMRIVRPGSVVGPQQQFMYMKQLEWAKWAAIDEIQRAEAQRVEVP--    364
P.striiformis         IYKYRFTAEEAVGFMRIMRPGTCVGPQQHFLYENQLTWIEWSARDELLAEQQAQISPSKT    368
                      :: : *:* * :.::*::***  *****:::: :   : :*
S.cerevisiae          LISL---EEY---------------RLQKKKLK----DDKRVAQNNIEGELRDLTMTPPS    396
C.zeina               TRLQ------------------------------------QSNSRKLAS--NGQTFTPPN    440
A.niger               -PIT---PG---------------------RPT----TRQR-------A--NGPVATPPN    419
B.cinerea             VTPR---KG---------------------HYT-------------------SKSQVVTP    426
M.oryzae              NTAN---TG---------------------VPS----TPIRAMQKASLG--RTSATQAST    438
C.gloeosporioides     ---N---AA---------------------VPS----TPIRAMQKTSLR--NGQASTPPH    425
U.maydis              KLEAATAAARAAAAAAEAANAATAAVALPMIPTTRASTKRRAAGSDAAKCAEADRFDQPD    526
R.solani              -TPRTP-PE----------------EIQPMTTGSTNGTSSSAVRSGNSAAPSSPRATTPP    432
P.striiformis         ERPITPPPE----------------NLAPGITIS------------------STTSTPPP    422

S.cerevisiae          NGHG--------A----LSARNSSQP---STAN---------------------------    414
C.zeina               GDRH--------Q----TSPRRALGE---ITNNEGA------------------------    461
A.niger               N-----------G----HSKRAALGE---IDHNEG-------------------------    436
B.cinerea             P-----------N----GNQRTPLGE---VDNERS-------------------------    443
M.oryzae              P-----------P----SKSRTPLGE---MDTES--------------------------    454
C.gloeosporioides     R-----------S----TSNRTPLSE---VDHDR--------------------------    441
U.maydis              ADVSLDEHIKHAPQLSRNTRRKPTSEHSDSDDDEGELQRPRQKRKSAAAAAAAAAAAAAA    586
R.solani              G-------LMSSPVI---------------------------------------------    440
P.striiformis         K-------SHMAPTM---------------------------------------------    430

S.cerevisiae          ----------------------NGSNSFKSSAVPQTSPGQPRKGQNGSNTIEDIN    447
C.zeina               PASTYTD-------------HMKSSVGVADENLPAPTPGQPRKTNKVYGGRR---    500
A.niger               -----------------------A-GTQAEEYLPAPTPGQPRKSHRKDSRHH---    464
B.cinerea             -----------------------HSIGAQEDYLPAPTPGQPRKTGRIDR--HH--    471
M.oryzae              -----------------------NTVGVQEDYLPAPTPGQPRKTGRNPSGRHH--    484
C.gloeosporioides     -----------------------NNIGAQEDYLPAPTPGQPRKTNRAGADRHH--    471
U.maydis              AAADYIDGSGPVTPIAQRASSRAVPASAAPVSKPAPCVGQPRKSPSPSRKR----    637
R.solani              -----NGGAVPVTPHKARP--------TTPIKT-DAPVLQPRKTPAAKRA-----    476
P.striiformis         -----PTSAVPG---------------------------QPRKTPGAKTRH----    449
                                                             ****
```

Figure S3

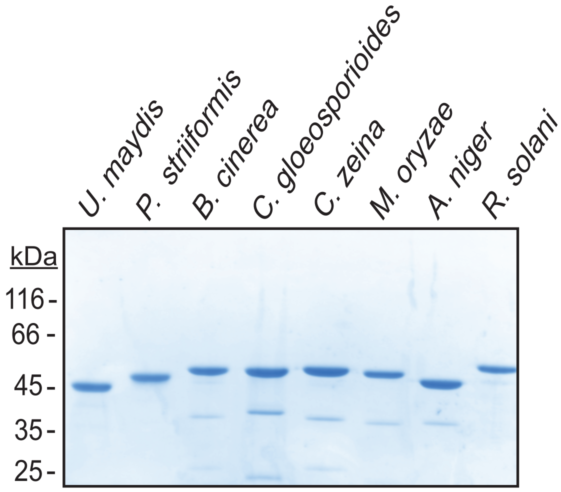
U. maydis
P. striiformis
B. cinerea
C. gloeosporioides
C. zeina
M. oryzae
A. niger
R. solani
kDa
116
66
45
35
25

Figure S4

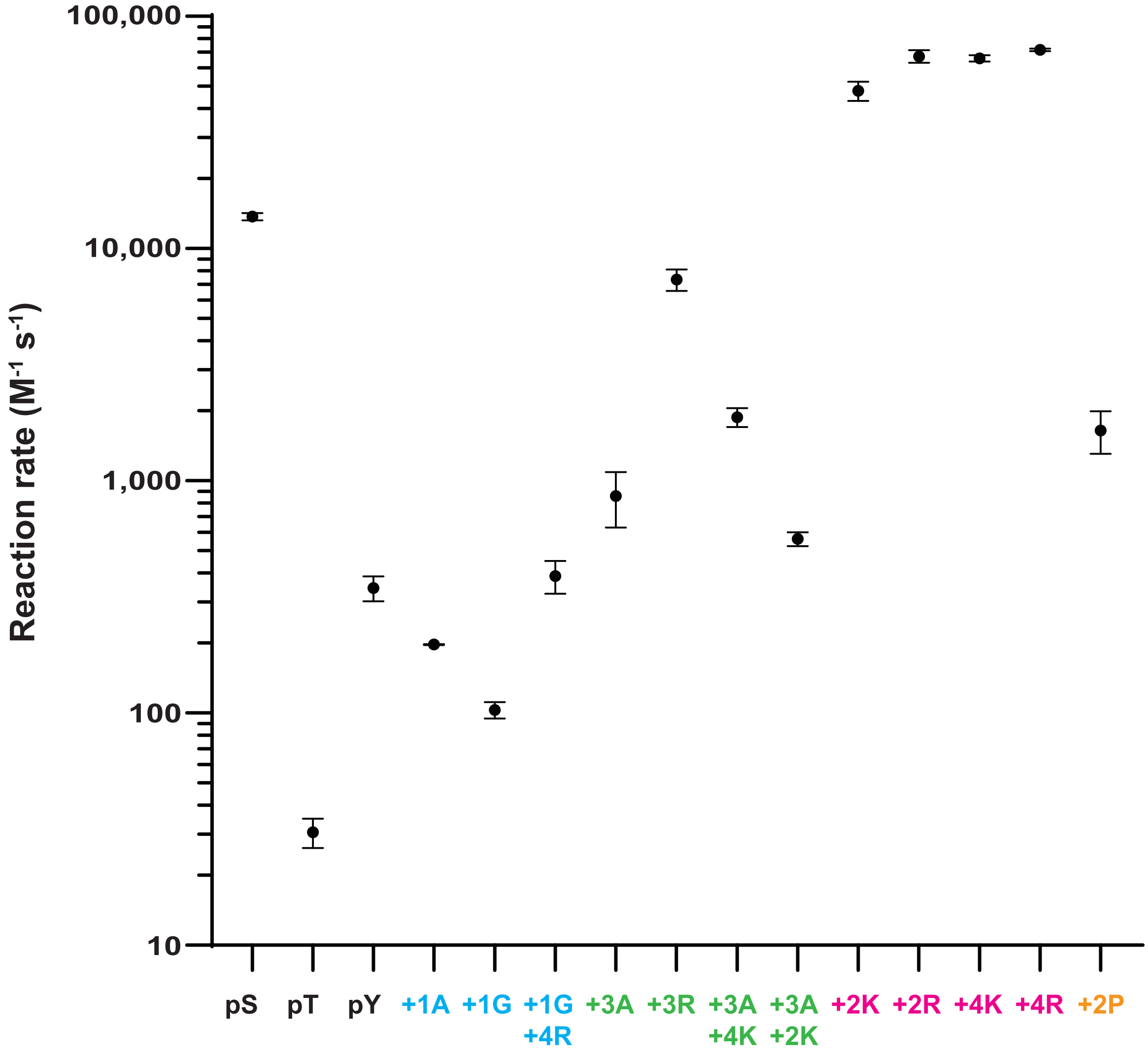

Reaction rate (M^{-1} s^{-1})
100,000
10,000
1,000
100
10
pS
pT
pY
+1A
+1G
+1G +4R
+3A
+3R
+3A +4K
+3A +2K
+2K
+2R
+4K
+4R
+2P
